# Prognostic Significance of the Metabolic Marker Hexokinase-2 in Various Solid Tumors: A Meta-Analysis

**DOI:** 10.1371/journal.pone.0166230

**Published:** 2016-11-08

**Authors:** Yulin Liu, Ke Wu, Liang Shi, Fan Xiang, Kaixiong Tao, Guobin Wang

**Affiliations:** 1 Department of Gastrointestinal Surgery, Union Hospital, Tongji Medical College, Huazhong University of Science and Technology, Wuhan, China; 2 Department of Clinical Laboratory, Union Hospital, Tongji Medical College, Huazhong University of Science and Technology, Wuhan, China; University of North Carolina at Chapel Hill School of Medicine, UNITED STATES

## Abstract

**Objective:**

Recently, numerous studies have reported that hexokinase-2 (HK2) is aberrantly expressed in cancer, indicating that HK2 plays a pivotal role in the development and progression of cancer. However, its prognostic significance in solid tumor remains unclear. Accordingly, we performed a meta-analysis to assess the prognostic value of HK2 in solid tumor.

**Methods:**

Eligible studies were identified using PubMed, Embase, and Web of Science databases. Pooled hazard ratios (HRs) with 95% confidence intervals (CIs) for overall survival (OS) or progression-free survival (PFS)/disease-free survival (DFS)/relapse-free survival (RFS) were estimated with random effects or fixed effects models, respectively. Subgroup analysis was also performed according to patients’ ethnicities, tumor types, detection methods, and analysis types.

**Results:**

Data from 21 included studies with 2532 patients were summarized. HK2 overexpression was significantly associated with worse OS (pooled HR = 1.90, 95% CI = 1.51–2.38, *p* < 0.001) and PFS (pooled HR = 2.91, 95% CI = 2.02–4.22, *p* < 0.001) in solid tumor. As to a specific form of cancer, the negative effect of HK2 on OS was observed in hepatocellular carcinoma (pooled HR = 2.06, 95% CI = 1.67–2.54, *p* < 0.001), gastric cancer (pooled HR = 1.72, 95% CI = 1.09–2.71, *p* = 0.020), colorectal cancer (pooled HR = 2.89, 95% CI = 1.62–5.16, *p* < 0.001), but not in pancreatic cancer (pooled HR = 1.13, 95% CI = 0.28–4.66, *p* = 0.864). No publication bias was found in the included studies for OS (Begg’s test, *p* = 0.325; Egger’s test, *p* = 0.441).

**Conclusion:**

In this meta-analysis, we identified that elevated HK2 expression was significantly associated with shorter OS and PFS in patients with solid tumor, but the association varies according to cancer type.

## Introduction

Cancer has being the leading cause of mortality worldwide and is therefore a major public health threat. Solid tumors account for most of human cancers [1]. Currently, curative surgical resection is still the optimal therapy for primary solid tumor. However, outcome may vary in patients with the same tumor after surgery. Thus, to precisely evaluate the therapeutic effect and prognosis of patients who suffer from cancer, feasible and effective biomarkers are urgently needed.

Numerous studies have shown that cancer metabolism is closely related to cancer cell survival owing to the sharing of common signaling molecules between these two pathways [2–4]. Cancer cells prefer to convert glucose to lactic acid as the main source of energy, regardless of oxygen availability, a phenomenon designated the Warburg effect or aerobic glycolysis [5]. Aerobic glycolysis generates less ATP than oxidative phosphorylation (OXPHOS), but can rapidly provide sufficient energy and biosynthetic precursors for cellular proliferation. Therefore aerobic glycolysis has been identified as the seventh hallmark of cancer [6]. Although the glycolysis rate can be regulated by a number of metabolic enzymes in the glycolytic pathway, most studies have suggested that glycolytic flux primarily relies on upregulation of glucose transporters and hexokinase (HK) [7, 8]. Specifically, HK2 has been suggested to contribute to the increased glycolysis by catalyzing the conversion from glucose to glucose-6-phosphate in the glycolytic pathway of cancer cells [9].

Recently, overexpression of HK2 has been reported in various solid tumors, including colorectal cancer [10], hepatocellular carcinoma [11], ovarian cancer [12], and pancreatic cancer [13], indicating that HK2 plays an important role in the development and progression of cancer. However, the prognostic value of HK2 was still controversial. Many studies have shown that overexpression of HK2 is associated with poor prognosis [14–16], whereas a few studies have demonstrated the opposite results [10].

Thus, in order to clarify the prognostic significance of HK2 as a metabolic marker in various solid tumors, we performed a meta-analysis on data collected systematically.

## Methods

### Search Strategy

The PubMed, Embase, and Web of Science databases were used for a comprehensive literature search with the following terms and their combinations: “hexokinase-2 or HK2 or HK-2”, “cancer or carcinoma or tumor or neoplasm”, and “prognosis or prognostic or outcome or survival”. The last search was conducted on September 28, 2016. Any potential studies were manually searched for in the references.

### Inclusion and Exclusion Criteria

Eligible studies met the following inclusion criteria: evaluation of patients with pathologically diagnosed solid tumors; the study contained sample size more than 20; published in English; studies aimed to explore the association between HK2 expression and prognosis; and studies with hazard ratios (HRs) and confidence intervals (CIs), which could be directly obtained or calculated with relevant data.

The exclusion criteria were as follows: studies of tumor cell lines, animal experiments, or non-solid tumors; published in a language other than English; duplicate reports and inappropriate article types, such as case reports, letters, conference papers, and reviews; studies without sufficient data for obtaining HRs and CIs.

### Data Extraction and Quality Assessment

Data were extracted from the included studies by two authors (Yulin Liu and Liang Shi) independently, and a third author (Ke Wu) was responsible for reconciling disagreements. Extracted data included first author’s name, publication year, country, tumor type, sample size, tumor stage, mean age, cut-off value, and outcome. The adopted indexes for outcomes included overall survival (OS) and progression-free survival (PFS)/disease-free survival (DFS)/relapse-free survival (RFS). The quality of the included studies was assessed using the Newcastle-Ottawa Scale (case-control studies) [17]. The score ranged from 0 to 9, and studies with scores of 6 or more were regarded as high quality.

### Statistical Analysis

HRs and their 95% CIs were used to assess the prognostic value of elevated HK2 expression in solid tumor. The reported statistical variables were extracted directly from the primary studies. If not given explicitly, variables were calculated using the Kaplan-Meier method or available data according to Tierney’s method [18]. Heterogeneity analysis was performed by visual observation of forest plots with the *I*^2^ statistic and Chi-squared tests [19]. *I*^2^ > 50% and/or *p* < 0.05 was considered representative of obviously heterogeneity, and a random effect model was applied to pool the HRs and their CIs. Otherwise, a fixed effect model was applied. Pooled HRs of greater than 1 represented poor prognosis of elevated HK2 expression, and results with *p* values of less than 0.05 were considered statistically significant. Subgroup analysis was further performed for interpretation of identified heterogeneity. Additionally, the consistency of pooled outcomes was examined with sensitivity analysis. Publication bias was investigated using funnel plots with Egger’s test and Begg’s test, and results with *p* values of less than 0.05 were considered to be indicative of significant bias. Stata 12.0 software was used for all statistical analyses in this meta-analysis.

## Results

### Eligible Studies

**Fig 1:**
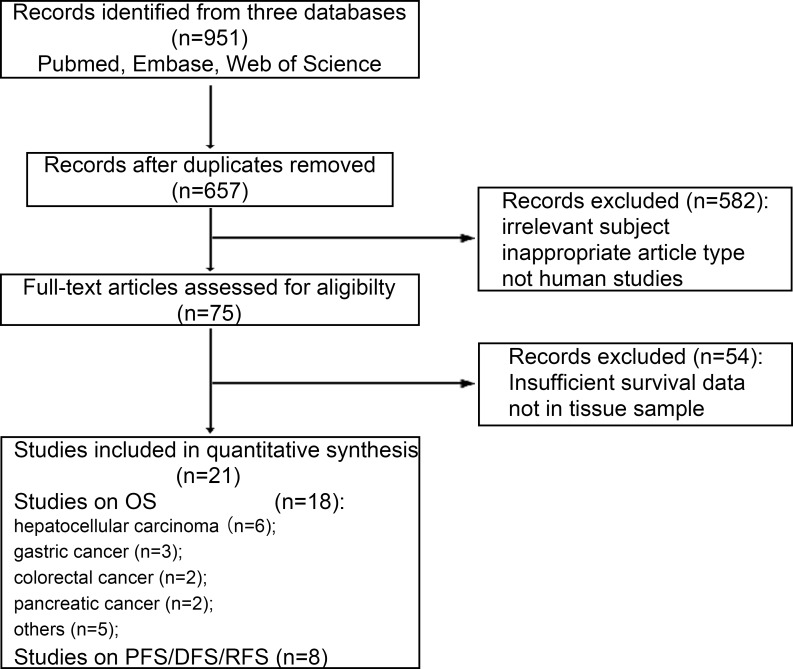
Flow diagram shows search strategy.

According to our defined searching strategy, a total of 951 references were identified after an initial search. After screening by titles and abstracts, 876studies were excluded on account of duplicated data, irrelevant subject, inappropriate article type, and inappropriate study type (i.e., cell and animal experiments). The 75 remaining studies for the association of HK2 overexpression with cancer prognosis were evaluated by reading the full text. Finally, 21studies consisting of 2532 cases were evaluated in this meta-analysis (Fig 1).

### Characteristics of Included Studies

The main characteristics of the included studies are shown in Table 1. Twenty-one eligible studies from China, Japan, Korea, the United States of America, Canada, and Germany included patients who had been diagnosed with a variety of solid tumors, such as hepatocellular carcinoma, gastric cancer, colorectal cancer and pancreatic cancer. HK2 protein and mRNA expression in tumor tissues were detected in 18 and three studies, respectively. 18 studies provided data on OS, which including hepatocellular carcinoma (n = 6), gastric cancer (n = 3), colorectal cancer (n = 2), pancreatic cancer (n = 2) and others (n = 5). Eight studies provided data on PFS/DFS/RFS. HRs were directly extracted from 14 studies and calculated with available data or survival curves from the other seven studies. The cut-off values for positive expression of HK2 varied in these studies. In quality assessment, all of the included studies had scores ranging from 6 to 9, indicating that they were of high quality.

**Table 1:** The main characteristics of the included studies in the meta-analysis.

First author	Year	Country	Tumor type	Sample size	Stage	Method	Mean age	Cut-off value	Analysis	Outcome	NOS
**Zhang [20]**	2016	China	HCC	155	I-IV	IHC	NR	CS	univariate	OS	7
**Guo [21]**	2015	China	HCC	120	I-IV	RT-PCR	NR	NR	survival curves	OS	7
**Gong [22]**	2012	China	HCC	97	I-IV	RT-PCR	NR	0.39%	survival curve	OS	8
**Kwee [23]**	2012	America	HCC	157	I-IV	IHC	NR	CS	multivariate	OS	9
**Peng [14]**	2008	China	HCC	203	I-IV	RT-PCR	54.9	ratio ≥1.5	survival curve	OS	8
**Paudyal [24]**	2008	Japan	HCC	31	I-IV	IHC	NR	CS	multivariate	OS	8
**Hur [25]**	2013	Korea	GC	152	I-IV	IHC	55.8±13.6	staining≥30%	univariate	OS/DFS	7
**Qiu [16]**	2011	China	GC	188	I-IV	IHC	57	NR	multivariate	OS	8
**Rho [26]**	2007	Korea	GC	257	I-IV	IHC	54.6	staining≥10%	multivariate	OS	9
**Katagiri [27]**	2016	Japan	CRC	195	I-IV	IHC	64.5	staining≥10%	multivariate	OS	8
**Ho [28]**	2016	Canada	CRC	60	I-IV	IF	NR	F-Score ≥ 24.7	univariate	OS/PFS	7
**Hamabe [10]**	2014	Japan	CRC	104	I-IV	IHC	NR	CS	survival curve	RFS	8
**Ogawa [13]**	2015	Japan	PC	36	I- III	IHC	70	CS	multivariate	OS/RFS	7
**Lyshchik [29]**	2007	Japan	PC	74	I-IV	IHC	63.5 ± 9.2	index≥3	survival curve	OS	8
**Sato [30]**	2013	Japan	BC	118	I- III	IHC	57	staining≥10%	survival curve	DFS	7
**Palmieri [31]**	2009	Germany	BC	123	NR	IHC	51	NR	multivariate	OS	8
**Zhang [32]**	2016	China	NPC	140	I-IV	IHC	48	index≥3	multivariate	OS/PFS	9
**Huang [33]**	2015	China	CCA	132	I-IV	IHC	51	staining≥25%	multivariate	PFS	8
**Suh[12]**	2014	Korea	EOC	111	I-IV	IHC	NR	CS	multivariate	OS/PFS	9
**Tsukada [34]**	2012	Japan	ULMS	23	I	IHC	51.5	CS	multivariate	OS	8
**Wolf [15]**	2011	Canada	GBM	56	NR	IHC	NR	NR	multivariate	OS	7

HCC: hepatocellular carcinoma; GC: gastric cancer; CRC: colorectal cancer; PC: pancreatic cancer; BC: breast cancer; NPS: nasopharyngeal carcinoma; CCA: cervical carcinoma; EOC: epithelial ovarian carcinoma; ULMS: uterine leiomyosarcoma; GBM: glioblastoma multiforme; IHC: immunohistochemistry; RT-PCR: reverse transcription-polymerase chain reaction; IF: immunofluorescence; NR: not reported; CS: complex score combining intensity and percentage of HK2 expression.

### Correlation of HK2 Expression with OS and Subgroup Analysis

**Fig 2:**
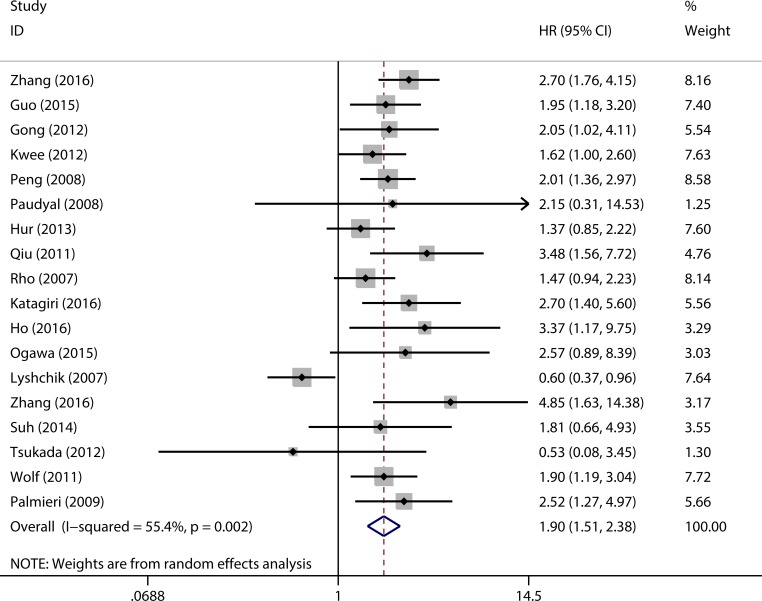
Forest plots to assess the effect of elevated HK2 expression on OS in patients with various solid tumors.

The combined analysis of 18 studies showed that elevated HK2 expression was associated with worse OS (pooled HR = 1.90, 95% CI = 1.51–2.38, *p* < 0.001) (Fig 2). Owing to moderate heterogeneity (*I*^*2*^ = 55.4%, *p* = 0.002), a random effect model was used to pool HRs.

In order to identify the possible sources of heterogeneity across these studies, subgroup analysis was performed according to patients’ ethnicity, tumor type, detection method, and analysis type (Table 2). Subgroup analysis by patient ethnicity showed that HK2 overexpression was significantly associated with worse OS in Asian patients (pooled HR = 1.86, 95% CI = 1.39–2.49, *p* < 0.001) and Caucasian patients (pooled HR = 1.97, 95% CI = 1.47–2.63, *p* < 0.001). The negative effect of HK2 overexpression on OS was demonstrated in patients with hepatocellular carcinoma (pooled HR = 2.06, 95% CI = 1.67–2.54, *p* < 0.001), gastric cancer (pooled HR = 1.72, 95% CI = 1.09–2.71, *p* = 0.020), colorectal cancer (pooled HR = 2.89, 95% CI = 1.62–5.16, *p* < 0.001) and others (pooled HR = 2.15, 95% CI = 1.44–3.21, *p* < 0.001), but not with pancreatic cancer (pooled HR = 1.13, 95% CI = 0.28–4.66, *p* = 0.864). In subgroup analysis based on detection method, overexpression of HK2 could significantly indicate poor outcome in patients with solid tumor (IHC: pooled HR = 1.85, 95% CI = 1.37–2.49, *p* < 0.001; RT-PCR: pooled HR = 2.00, 95% CI = 1.51–2.65, *p* < 0.001). Subgroup analysis by the type of analysis suggested that elevated HK2 expression was significantly associated with shorter OS using either univariate analysis (pooled HR = 1.72, 95% CI = 1.13–2.61, *p* = 0.011) or multivariate analysis (pooled HR = 1.99, 95% CI = 1.60–2.46, *p* < 0.001).

**Table 2:** Pooled HRs for OS according to subgroup analysis.

Subgroup	No. of patients	No. of studies	Random-effect model		Heterogeneity	
			HR (95% CI)	*p* value	*I*^*2*^ (%)	*p* value
**Overall survival**	2178	18	1.90 (1.51, 2.38)	< 0.001	55.4	0.002
**Ethnicity**						
Asian	1782	14	1.86 (1.39, 2.49)	< 0.001	63.4	0.001
Caucasian	396	4	1.97 (1.47, 2.63)	< 0.001	0	0.541
**Tumor type**						
HCC	763	6	2.06 (1.67, 2.54)	< 0.001	0	0.767
GC	597	3	1.72 (1.09, 2.71)	0.020	52.1	0.124
CRC	255	2	2.89 (1.62, 5.16)	< 0.001	0	0.731
PC	110	2	1.13 (0.28, 4.66)	**0.864**	81.7	0.019
Others	453	5	2.15 (1.44, 3.21)	< 0.001	17.6	0.303
**Detection method**						
IHC	1698	14	1.85 (1.37, 2.49)	< 0.001	63.9	0.001
RT-PCR	420	3	2.00 (1.51, 2.65)	< 0.001	0	0.992
IF	60	1	3.37 (1.17, 9.74)	0.024	-	-
**Analysis type**						
Multivariate	1317	11	1.99 (1.60, 2.46)	< 0.001	4.3	0.402
Univariate	861	7	1.72 (1.13, 2.61)	0.011	77.3	< 0.001
**Lyshchik's study**						
Yes	2178	18	1.90 (1.51, 2.38)	< 0.001	55.4	0.002
No	2104	17	2.00 (1.73, 2.32)	< 0.001	0	0.475

### Correlation of HK2 Expression with PFS/DFS/RFS

**Fig 3:**
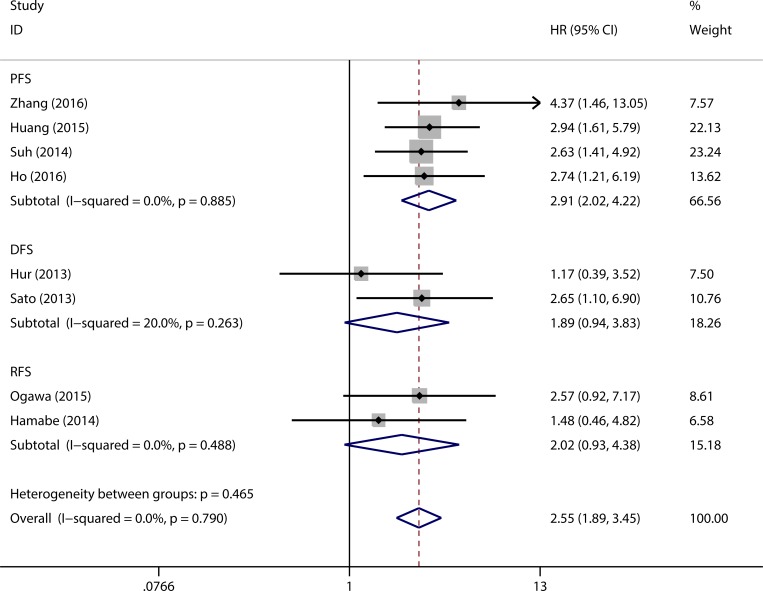
Forest plots to assess the effect of elevated HK2 expression on PFS/DFS/RFS.

Eight eligible studies were adopted to pool HRs for PFS/DFS/RFS. Without obvious statistical heterogeneity (*I*^*2*^ = 0.0%, *p* = 0.790), a fixed effect model was used to pool HRs. The results showed that elevated HK2 expression was associated with negative outcome in patients with solid tumor (pooled HR = 2.55, 95% CI = 1.89–3.45, *p* < 0.001) (Fig 3). Additionally, data were analyzed based on PFS, DFS, and RFS. Patients with elevated HK2 expression had a significantly shorter PFS (pooled HR = 2.91, 95% CI = 2.02–4.22, *p* < 0.001). Despite the lack of significant difference, a similar trend was observed for DFS (pooled HR = 1.89, 95% CI = 0.94–3.83, *p* = 0.074) and RFS (pooled HR = 2.02, 95% CI = 0.93–4.38, *p* = 0.076).

### Sensitivity Analysis

**Fig 4:**
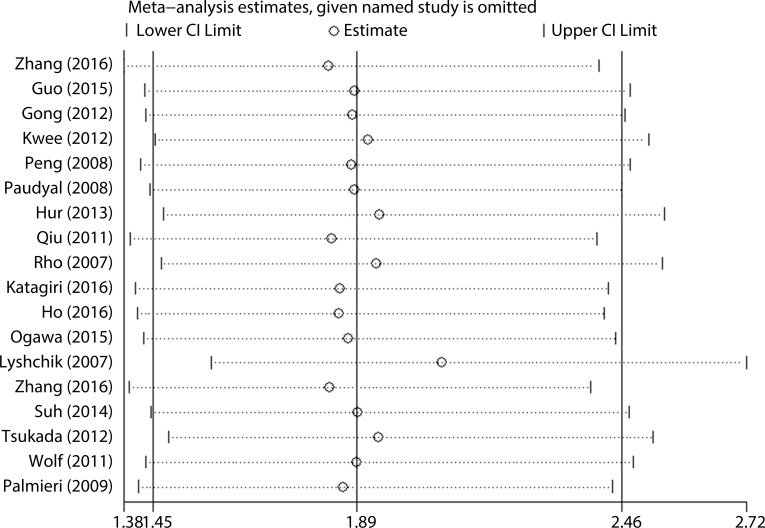
Sensitivity analysis of the evaluation on the relationship between HK2 expression and OS.

Sensitivity analysis was performed by sequentially omitting individual studies with a fixed-effect model (Fig 4). No studies were found to obviously impact the result pattern. Interestingly, one study by Lyshchik et al [22] contributed the most to the observed heterogeneity. After deletion of this study, the observed heterogeneity dramatically decreased (*I*^*2*^ = 55.4%, *p* = 0.002 versus *I*^*2*^ = 0.0%, *p* = 0.475). However, the association between HK2 expression and OS was not altered when this study was included (pooled HR = 1.90, 95% CI = 1.51–2.38, *p* < 0.001) or excluded (pooled HR = 2.00, 95% CI = 1.73–2.32, *p* < 0.001).

### Publication Bias

**Fig 5:**
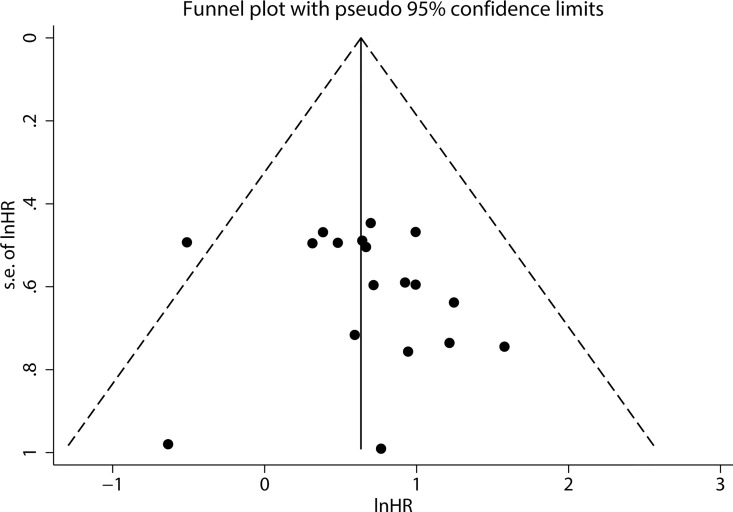
Funnel plots to evaluate publication bias of included studies for OS.

Publication bias was not found by visual assessment of funnel plots (Fig 5), and this analysis was confirmed by Begg’s test (*p* = 0.325) and Egger’s test (*p* = 0.441). Because fewer than 10 eligible studies were used to pool HRs for PFS/DFS/RFS, funnel plots, Begg’s tests, and Egger’s tests were not performed and publication bias was not assessed in these analyses.

## Discussion

In the past several decades, aerobic glycolysis has attracted increasing attention as a hallmark of cancer cells. Metabolic reprogramming furnishes cancer cells with sufficient precursor substances for rapid cell proliferation [35, 36]. As a key irreversible step in the glycolytic pathway, the phosphorylation of glucose catalyzed by HK plays a pivotal role in aerobic glycolysis. Meanwhile, HK is involved in the initiation and progression of tumor; in particular, HK2 is a critical component integrating cell viability and energy production pathways [21, 37]. The pro-survival effect of HK2 is achieved mainly through the following three mechanisms: (1) upregulation of HK2 facilitates the generation of biosynthetic precursors for cellular proliferation by increasing glycolysis rate [38]; (2) overexpression of HK2 could increase NADPH levels in the cytoplasm through the pentose phosphate pathway, contributing to protection of cells from oxidative damage [39]; and (3) HK2 can bind to mitochondria and preserve mitochondrial integrity in order to block mitochondrial death pathways [40]. Therefore, elevated expression of HK2 is critical for promoting tumor progression.

Accumulating evidence has revealed that elevated HK2 expression is associated with not only the development and progression of tumor [37, 41], but also with an unfavorable prognosis [23]. The overexpression of HK2 was positively correlated with tumor stage, differentiation and lymph node metastasis, as well as with a reduction in survival [10, 16, 21, 32].To the best of our knowledge, the present study is the first meta-analysis systematically exploring the prognostic value of elevated HK2 expression in solid tumor. In brief, our meta-analysis provided strong support that elevated HK2 expression was a significant independent indicator of poor outcome based on pooled HRs estimates. HK2 was associated with shorter OS in hepatocellular carcinoma, gastric cancer and colorectal cancer. The adverse prognostic effect of HK2 overexpression was virtually unaffected by ethnic background, analysis type and detection method. Moreover, patients with elevated HK2 expression had a significantly shorter PFS. Besides as an independent indicator of poor prognosis, combination of the expression of HK2 and other biomarkers could hold potent prognostic value in patients with solid tumor. In pancreatic cancer, although the positive expression of HK2 exhibited borderline significant prognostic value for OS, the combination of elevated expression of HK2 and PKM2 was found to be significant (P<0.05) [13]. Hamabe et al. reported that the combined evaluation of positive HK2 and negative phosphorylated pyruvate dehydrogenase (p-PDH) was associated with reduced RFS in stage II and III patients with CRC [10].[29]

Interestingly, we also found HK2 failed to predict the outcome of patients with pancreatic cancer. This discrepancy may be partly attributed to the small sample size in the individual studies [13, 29]. In addition, data from univariate analysis were adopted to estimate the prognostic role of HK2 expression in the study by Lyshchik et al [29], allowing us to ignore the effects of clinicopathological factors on the prediction efficacy of HK2. Sensitivity analysis revealed that the study by Lyshchik et al was the main source of heterogeneity. After deletion of this study, the observed heterogeneity completely disappeared. Finally, considering that pancreatic cancer remains a lather disease in spite of improvement in early diagnosis, curative surgical resection, and adjuvant therapies, multidisciplinary treatment is required for a complete cure [42]. Preoperative treatment have an effect on immunohistochemical staining patterns, which may impair the predictive efficacy of HK2 on outcome in tumor.

And yet for all that, as a key regulator of glycolysis, the expression of HK2 is likely significant for the progression and prognosis of pancreatic cancer. The dense desmoplastic region surrounding pancreatic cancer cells inhibits neovascularization, leading to the deficiency of oxygen and nutrients in tumor tissue [43, 44]. Under such stressful microenvironment, cancer cells undergo a shift in cellular metabolism from oxidative phosphorylation to glycolysis [45], as a hallmark of cancer cells. Therefore, more studies designed rationally are required to confirm that elevated HK2 expression is associated with unfavorable outcome in patients with pancreatic cancer.

In spite of the strong results of our study, there were still several limitations to our meta-analysis. First, although the study by Lyshchik et al was identified as the main source of heterogeneity, specific clinical elements that contributed to the heterogeneity were not explicit. Second, due to the use of various cut-off values in the included studies, the utility of HK2 as a prognostic biomarker was impaired in patients with solid tumor. Accordingly, it may be necessary to standardize detection methods and cut-off values. Third, because multiple factors affect prognosis, we preferred to include studies in which multivariate analysis had been performed; however, eight studies with univariate analysis only were still included for a comprehensive analysis. In general, multivariate analysis is more efficient and precise when there are multiple interacting variables in one experiment [46]. Subgroup analysis indicated that multivariate analysis also yielded more stable results. Finally, the approach for obtaining essential data to pool HRs could be a potential source of heterogeneity. The pooled HRs extracted directly from the primary studies seemed to be more reliable than those calculated with survival curves or available data. Although subgroup analysis by tumor type was conducted, the impact of HK2 on prognosis was lost in some specific forms of cancer. Hence, it is essential for more studies to enroll patients with specific forms of cancer.

In conclusion, our meta-analysis suggested that HK2 could act as an independent prognostic factor for patients with solid tumor, but the association between HK2 and prognosis varies according to cancer type. Based on the above-mentioned limitations, this conclusion should be further confirmed by additional clinical studies with larger sample sizes.

## Supporting Information

S1 Fig Forest plots to assess the association between elevated HK2 expression and OS in subgroups. (TIF)

S2 Fig Forest plot to assess the effect of elevated HK2 expression on OS after omitting Lyshchik’s study. (TIF)

S1 Table PRISMA 2009 checklist. (DOC)
