# Supplementary figures and images for: Prognostic Significance of the Metabolic Marker Hexokinase-2 in Various Solid Tumors: A Meta-Analysis
Source: PLoS One. 2016 Nov 8;11(11):e0166230. doi: 10.1371/journal.pone.0166230 (PMC5100994; doi:10.1371/journal.pone.0166230)

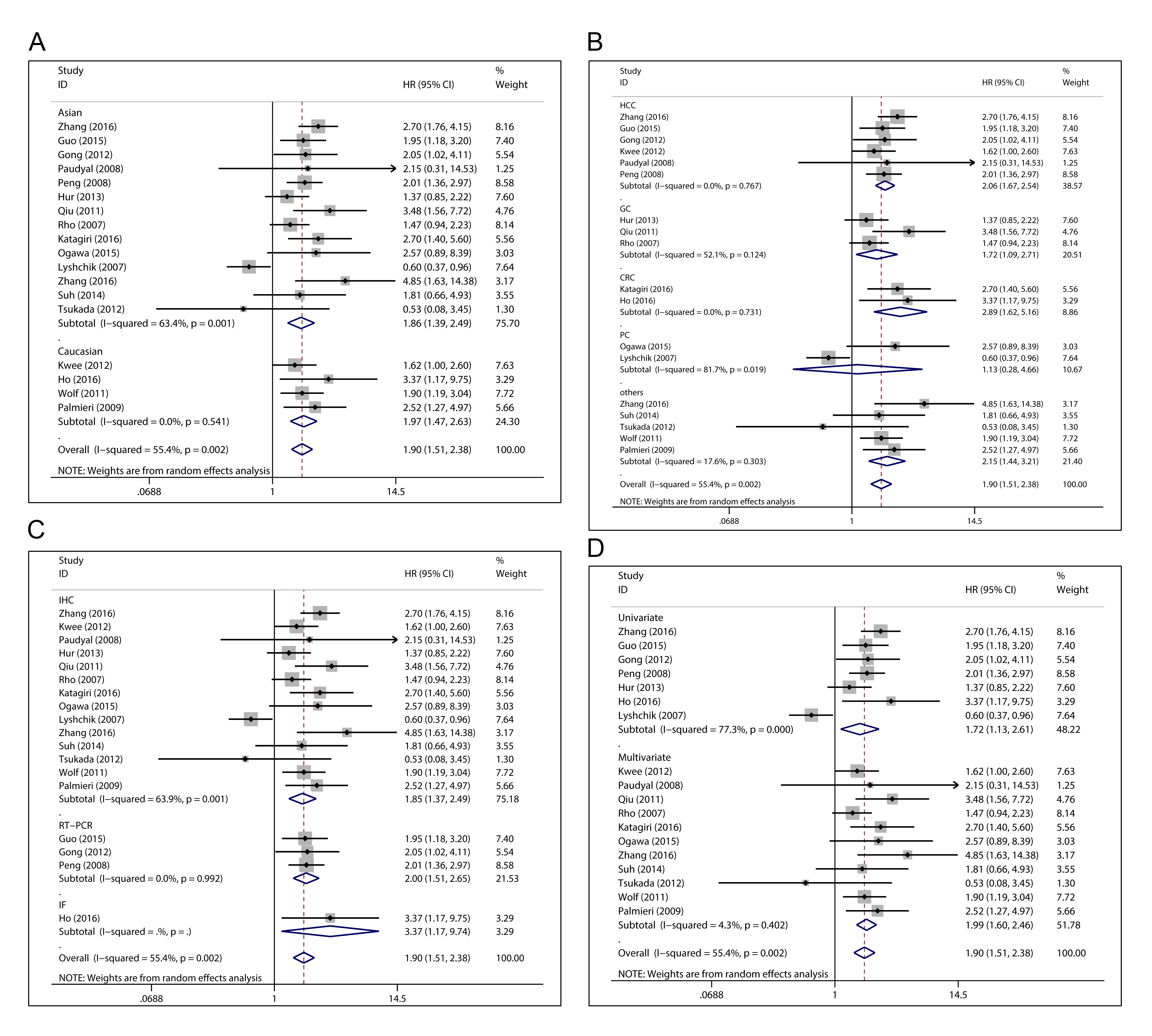

Supplement: S1 Fig — (TIF) [file pone.0166230.s001.tif]

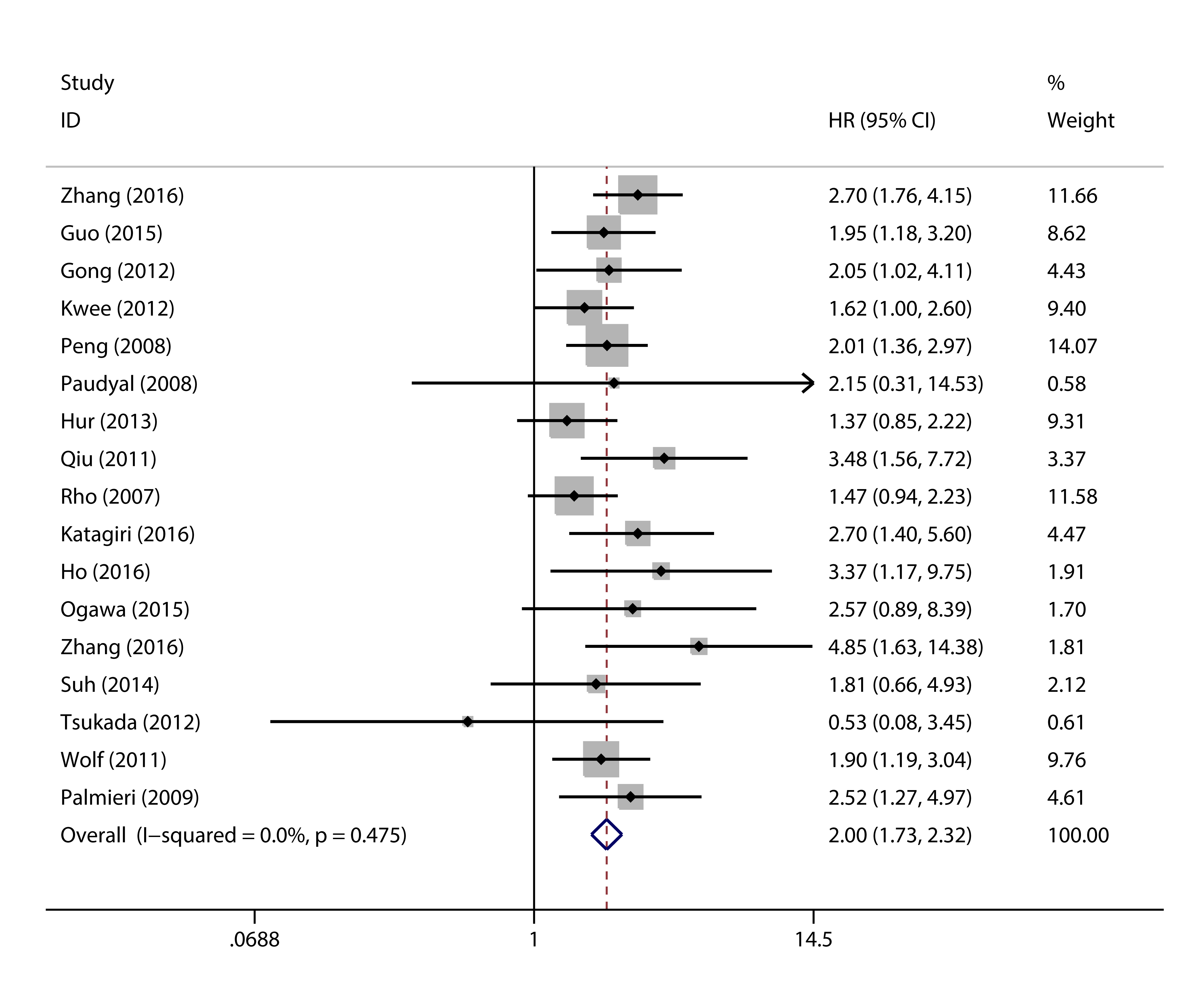

Supplement: S2 Fig — (TIF) [file pone.0166230.s002.tif]
